# Regulation of T_H_17 Cells and Associated Cytokines in Wound Healing, Tissue Regeneration, and Carcinogenesis

**DOI:** 10.3390/ijms18051033

**Published:** 2017-05-11

**Authors:** Leonie Brockmann, Anastasios D. Giannou, Nicola Gagliani, Samuel Huber

**Affiliations:** 1 I. Department of Medicine, University Medical Center Hamburg-Eppendorf, 20246 Hamburg, Germany; l.brockmann@uke.de (L.B.); a.giannou@uke.de (A.D.G.); n.gagliani@uke.de (N.G.); 2 Department of General, Visceral and Thoracic Surgery, University Medical Center Hamburg-Eppendorf, 20246 Hamburg, Germany; 3 Department of Medicine Solna (MedS), Karolinska Institute, 17177 Stochkolm, Sweden

**Keywords:** T_H_17 cells, cytokines, wound healing, tissue regeneration, carcinogenesis, immune regulation

## Abstract

Wound healing is a crucial process which protects our body against permanent damage and invasive infectious agents. Upon tissue damage, inflammation is an early event which is orchestrated by a multitude of innate and adaptive immune cell subsets including T_H_17 cells. T_H_17 cells and T_H_17 cell associated cytokines can impact wound healing positively by clearing pathogens and modulating mucosal surfaces and epithelial cells. Injury of the gut mucosa can cause fast expansion of T_H_17 cells and their induction from naïve T cells through Interleukin (IL)-6, TGF-β, and IL-1β signaling. T_H_17 cells produce various cytokines, such as tumor necrosis factor (TNF)-α, IL-17, and IL-22, which can promote cell survival and proliferation and thus tissue regeneration in several organs including the skin, the intestine, and the liver. However, T_H_17 cells are also potentially pathogenic if not tightly controlled. Failure of these control mechanisms can result in chronic inflammatory conditions, such as Inflammatory Bowel Disease (IBD), and can ultimately promote carcinogenesis. Therefore, there are several mechanisms which control T_H_17 cells. One control mechanism is the regulation of T_H_17 cells via regulatory T cells and IL-10. This mechanism is especially important in the intestine to terminate immune responses and maintain homeostasis. Furthermore, T_H_17 cells have the potential to convert from a pro-inflammatory phenotype to an anti-inflammatory phenotype by changing their cytokine profile and acquiring IL-10 production, thereby limiting their own pathological potential. Finally, IL-22, a signature cytokine of T_H_17 cells, can be controlled by an endogenous soluble inhibitory receptor, Interleukin 22 binding protein (IL-22BP). During tissue injury, the production of IL-22 by T_H_17 cells is upregulated in order to promote tissue regeneration. To limit the regenerative program, which could promote carcinogenesis, IL-22BP is upregulated during the later phase of regeneration in order to terminate the effects of IL-22. This delicate balance secures the beneficial effects of IL-22 and prevents its potential pathogenicity. An important future goal is to understand the precise mechanisms underlying the regulation of T_H_17 cells during inflammation, wound healing, and carcinogenesis in order to design targeted therapies for a variety of diseases including infections, cancer, and immune mediated inflammatory disease.

## 1. Inflammation in Wound Healing and Carcinogenesis

In 1986, Dvorak published an essay with the vivid title: “Tumors: Wounds that do not heal”, summarizing in this one statement the relation between wound healing and carcinogenesis [1]. Wound healing normally follows sequential but overlapping steps. The immediate reaction is hemostasis to provisionally close the wound. A fibrin clot is formed and platelets aggregate. This is followed by the inflammatory phase which is characterized by the presence of neutrophils, macrophages, and lymphocytes in the wound. These cells are attracted by chemokines, which are, for example, released by platelet cells [2]. The inflammatory phase is important for tissue regeneration due to the release of pro-inflammatory cytokines and growth factors from immune cells [3,4,5]. Additionally, phagocytes can ingest cell debris and invading pathogens. Therefore, the inflammatory phase is essential to prevent spreading of infections. The proliferative phase follows several days later leading to re-epithelialization, formation of new blood vessels, and fibrogenesis. During the last phase, the resolution phase, vessel regression and collagen remodeling occur [6]. Thus, occurrence of a wound has a dramatic impact on the body. Multiple cell types are necessary to secure a prompt healing process. Research elucidating this process mainly focuses on innate immunity contributing to tissue regeneration. However, adaptive immunity also plays its part during this process, even though its contribution and regulation is much less understood.

Tissue damage, especially at barrier organs such as the intestine, the lung, and the skin, is a potential gateway for invading pathogens, therefore inflammation is an essential part of wound healing. In this regard, the involvement of T cells during wound repair has been under investigation for a long time. In 1987, the hypothesis that T lymphocytes represent the most frequent leucocyte population in skin wounds was published [7]. Several studies indicate that delayed infiltration of T cells and a lower concentration of these cells at the site of the wound are associated with impaired wound healing. Furthermore, CD4^+^ T cells seem to play a beneficial role during the process of wound healing and regeneration [8,9,10]. However, very little is known about the contribution of different T cell subsets. Furthermore, these mechanisms, which are designed to promote wound healing, also have the potential to promote chronic inflammation and carcinogenesis. Two important predisposing factors for colorectal carcinogenesis are chronic intestinal inflammation and tissue injury. This association is based on the fact that wound healing and carcinogenesis are driven by several common factors and signaling pathways. Upon tissue injury, factors promoting healing are stimulated and their action must be tightly controlled in order to avoid carcinogenesis [11]. A chronically inflamed and wounded tissue is associated with a long-lasting healing response which may lead to fibrosis, tissue dysfunction, and ultimately the development of cancer. Thus, carcinogenesis could be considered as a consequence of failing regulatory mechanisms allowing abnormal excessive healing [11].

## 2. CD4^+^ T Helper Cells

Conventional αβ CD4^+^ T cells are one of the main players during an adaptive immune response. Due to their great variety, CD4^+^ T cells can orchestrate the immune response and react to the whole spectrum of immune challenges. Two major CD4^+^ T helper cell subsets, T_H_1 and T_H_2, were discovered in 1989 [12]. However, the T_H_1/T_H_2 paradigm was challenged in 2005 by the description of T_H_17 cells, which are primarily needed for the defense against extracellular bacteria and fungi [13]. Additionally, T_H_17 cell associated cytokines can promote epithelial proliferation and tissue regeneration [14].

The gastrointestinal tract, like skin and lung, is constantly in contact with hundreds of different species of commensal bacteria and fungi [15]. How the commensal microbiota modulate the immune system of the host and vice versa has been an area of intensive research and is still not completely understood. Nonetheless, it is known that the induction of T_H_17 cells is dependent on the presence of microbiota, especially segmented filamentous bacteria (SFB) in the terminal ileum of the small intestine in mice. Hence, under physiological conditions, T_H_17 cells are mainly located in the small intestine [16]. Upon tissue damage, T_H_17 cells rapidly expand at mucosal surfaces to guarantee a quick clearance of the invading microorganisms such as commensal bacteria. To this end, T_H_17 cells release several chemokines and cytokines, such as IL-17A, IL-17F, and IL-22 which are the signature cytokines of T_H_17 cells. One major defense mechanism of T_H_17 cells is the attraction of inflammatory cells via chemotaxis to the site of infection, which is crucial for the fast clearance of pathogens [17,18]. Additionally, T_H_17 cells contribute to the crosstalk of immune cells and epithelial cells or other innate immune cells by inducing the release of anti-microbial peptides [19,20,21,22]. The signature cytokines, IL-17A and IL-17F, both binding to IL-17RA [23], and IL-22, binding to IL-22R1, mediate this effect of T_H_17 cells. IL-22 does not only promotes the secretion of anti-microbial peptides from the epithelium, but also exhibits tissue protective properties. An important effect of IL-22 signaling on epithelial cells is the induction of proliferation, survival, and tissue repair via induction of STAT3 [14,24,25]. Thus, T_H_17 cells act at different front lines during the defense of the body: (a) T_H_17 cells activate and attract other immune cells, mainly neutrophils, which can phagocyte pathogens. Therefore, tissue resident T_H_17 cells could contribute to the inflammatory phase of wound healing. (b) T_H_17 cells induce the release of anti-microbial peptides from non-immune cells. (c) Finally, T_H_17 cell associated cytokines, such as IL-22, can promote wound healing and tissue regeneration, leading to a faster closing of potential entryways of microorganisms. In the next sections, we will give an overview of how T_H_17 cell associated cytokines affect wound healing and carcinogenesis.

## 3. Effects of T_H_17 Cell Associated Cytokines on Wound Healing and Carcinogenesis

### 3.1. Effects of IL-17 on Wound Healing

Interleukin-17 (IL-17), also known as IL-17A, is the main member of the IL-17 family, which consists of six members: IL-17A, IL-17B, IL-17C, IL-17D, IL-17E (also named IL-25), and IL-17F. This mediator is not only secreted by T_H_17 cells alone, but also by other immune cell types such as natural killer (NK), natural killer T (NKT), lymphoid tissue inducer (LTi), and LTi-like cells. Apart from immune cells, IL-17A can also be produced by Paneth cells which are epithelial cells located in the small intestine. To conduct its biological functions, IL-17 binds to a heteromeric receptor complex consisting of IL-17RA and IL-17RC. Along with three additional variants, namely IL-17RB, IL-17RD and IL-17RE, IL-17RA and IL-17RC, they comprise the IL-17 receptor family [26]. IL-17 is a pro-inflammatory cytokine, which is commonly found at high levels in inflamed sites. Recent studies have identified a link between the IL-23 induced expansion of T_H_17 cells followed by IL-17 secretion and the pathogenesis of IBD [27]. T_H_17 cells are mainly responsible for IL-17 production, which is elevated in IBD. Controversially, a role of IL-17 in IBD-related mucosal healing was recently found. Song et al. showed that following epithelial barrier damage, FGF2 is highly expressed and cooperates with IL-17 in order to enhance tissue repair. Specifically, upon barrier destruction, dysregulated microbiota cause an upregulation of TGF-β which subsequently stimulates FGF2 production by regulatory T cells. Then, FGF2 together with IL-17 upregulates genes associated with epithelial healing and boosts epithelial cell proliferation [28].

Recent studies focusing on skin wound healing have concluded that IL-17 plays a role in the early inflammatory stage of the wound and could act as an inhibitor to normal wound repair. As seen in psoriasis, IL-17 has several functions in the skin. The expression of IL-17 receptor on keratinocytes, fibroblasts, and inflammatory cells reveals that IL-17 can affect numerous cell types in the skin. In skin lesions, it is macrophages and not T cells which are the main cellular source of IL-17. To elucidate the role of IL-17 in skin wound healing, Rodero et al. used *Il17*^−/−^ mice and showed that absence of IL-17 enhances and accelerates skin tissue repair. Blocking IL-17 with specific antibodies during the early steps of healing also resulted in wound closure promotion [29]. On the other hand, a beneficial role of IL-17 in sensory nerve regeneration has been recently revealed. In a mouse model of corneal abrasion, an IL-17 dependent signaling pathway involving IL-17, neutrophils, platelets, and vascular endothelial growth factor (VEGF)-A was found to promote corneal repair and nerve regeneration [30].

In conclusion, IL-17 seems to have dual functions during wound healing. It may have beneficial functions in the intestine and for nerve regeneration [31]. However, in the skin, especially in psoriasis, it seems to be mainly pathogenic. Environmental factors may play a crucial role determining the part IL-17 has in wound healing, but further studies are needed to clarify this point.

Likewise, the role of IL-17 during carcinogenesis is still not completely understood. T_H_17-related events and specifically the role of IL-17 were first thought to promote tumor growth and invasion and to enhance angiogenesis [32]. However, other studies identified a protective role in tumor immune surveillance together with an inhibition of cancer cell proliferation and metastasis [33,34,35]. Using spontaneous intestinal carcinogenesis models, the role of IL-17A in colorectal cancer development was further elucidated. Recent studies showed that blocked or genetically-induced absence of IL-17A resulted in significantly attenuated tumor burden in Apc^Min/+^ mice infected with enterotoxigenic *Bacteroides fragilis* and in the standard Apc^Min/+^ model [36,37]. In the dextran sulfate sodium/azoxymethane (DSS/AOM) carcinogenesis model, *Il17a*^−/−^ mice displayed reduced tumorigenesis, which could be attributed to lower levels of intestinal TNF-α, interferon (IFN)-γ, IL-6 ,and STAT3 activity [38]. Interestingly, a highly expressed T_H_17 cell related mRNA pattern correlates with poor prognosis in human colorectal cancer [39,40]. This observation is likely associated with the fact that T_H_17 cells are the main IL-17A producers in the tumor. However, other immune cell subsets such as CD8^+^ CTLs (Tc17 cells), γδ T cells (γδT17 cells), and innate lymphoid cells (ILCs) also produce IL-17A [41,42,43]. Notably, upon dendritic cell (DC)-mediated stimulation, γδT17 cells produce IL-17 and enhance the recruitment and expansion of myeloid-derived suppressor cells. This finding suggests that IL-17A is likely to be associated with immune silencing in colorectal cancer. Due to the well-described implication of IL-17A in colorectal cancer, targeting of IL-17A may serve as a promising therapeutic approach. Interestingly, a recent study showed that blocking of IL-17A in an adenomatous polyposis coli (APC)-mediated colon carcinogenesis model enhanced tumor sensitivity to the anticancer agent 5-fluorouracil [44]. Similarly, antiangiogenic therapies might fail due to the IL-17A-driven emergence of resistant tumor stromal cells [45].

Apart from IL-17A, another member of the IL-17 superfamily and ligand of IL-17RE, IL-17C, was shown to be expressed in intestinal epithelial cells during early stages of colon carcinogenesis. In both the Apc^Min/+^ and the DSS/AOM-induced carcinogenesis models, mice lacking IL-17RE displayed a decreased tumor burden together with a lower expression of the anti-apoptotic proteins BCL-2 and BCLXL. Interestingly, IL-17C can be associated with the human condition, since it is overexpressed in human colon cancers [46]. IL-17F, which also belongs to the IL-17 family, resembles IL-17A and acts via the same receptor, and may play an opposite role in colon carcinogenesis. In contrast to IL-17A and C, IL-17F is significantly downregulated in human colorectal cancer. Notably, it reduces carcinogenesis in the DSS/AOM carcinogenesis model by inhibiting angiogenesis [47].

### 3.2. Effects of IL-22 on Wound Healing

Interleukin-22 (IL-22), a member of the IL-10 cytokine family, participates in the signaling between the immune system and the peripheral tissues. IL-22 can be produced by several other cell types such as T_H_22, T cell receptor (TCR)-γδ, NK, NKT, ILCs, and LTi cells, with the notable exception of T_H_17 cells. IL-22 acts via binding to the heterodimer IL-10R2/IL-22R1 complex [48]. IL-22R1 is expressed on non-hematopoietic cells, such as intestinal epithelial cells, hepatocytes, and fibroblasts in the skin. After binding of IL-22 to the receptor complex, STAT3, STAT1, and STAT5, as well as the Janus kinase (JNK) and mitogen-activated protein kinases are activated. The translocation of activated STAT dimers into the nucleus leads to the activation of several genes linked to proliferation and cell survival. IL-22 is known to play a key role in tissue regeneration and wound healing [3]. In a mouse model of acute skin injury, an upregulation of *Il22* mRNA expression was observed during the inflammatory stage and IL-22 was identified as a critical mediator for normal fibroblast function, extracellular matrix protein production, and myofibroblast differentiation during skin wound healing [49]. IL-22 was found to facilitate the crosstalk between immune cells and fibroblasts during skin wound healing. It has also been shown to promote keratinocyte proliferation and migration while acting as an inhibitor for keratinocyte differentiation [3,50,51,52]. IL-22 is unlikely to play a major role in the early stages of skin wound healing such as immune cell accumulation and angiogenesis. Similarly, loss of IL-22 does not affect keratinocyte function during skin wound healing. However, upon wound healing, fibroblast function was shown to be IL-22-dependent. Specifically, absence of IL-22 leads to impaired granulation, tissue formation, production of extracellular matrix components (ECM), and wound contraction. Primary dermal fibroblasts are directly affected by IL-22, since they express IL-22R1, whose IL-22-triggered activation can lead to STAT3 phosphorylation. IL-22 stimulates ECM production by inducing ECM gene expression in fibroblasts and by promoting myofibroblast differentiation. A decreased number of myofibroblasts in the wound may lead to defective wound contraction and impaired ECM formation, as seen in *Il22*^−/−^ mice. IL-22 may induce the expression of ECM genes, mainly via STAT3 activation. IL-22-mediated STAT3 phosphorylation leads to activation of the promoters of fibronectin and collagen.

Interestingly, IL-22 is also essential for intestinal healing and the maintenance of the mucosal barrier. In studies using the DSS-induced acute intestinal injury in *Il22*^−/−^ mice, IL-22 was shown to enhance intestinal wound healing, specifically via STAT3 activation, which in turn regulates signaling pathways commonly associated with tissue repair and gut homeostasis. Mice lacking IL-22 exhibited an impaired and delayed recovery from DSS-caused intestinal injury [14]. Similarly, targeting of IL-22 with specific neutralizing antibodies led to impaired wound healing in wildtype mice. On the other hand, increase of intestinal IL-22 expression via a gene delivery system boosted the recovery of the injured intestine [24]. Interestingly, another study showed that IL-22 deficiency leads to altered intestinal microbiota and therefore increases the severity of the disease in a mouse model of experimental colitis. This IL-22-mediated changed microbiota can be transferred to co-housed wild type mice, which subsequently become more susceptible to experimental colitis, suggesting that IL-22 is essential for maintaining the balance between immunity and intestinal microbiota [52].

Although the beneficial role of IL-22 in wound healing is well documented, in some cases the delicate balance between protection and harm shifts in favor of the pathogenic direction leading to carcinogenesis. A tumor-promoting function of IL-22 via STAT3 stimulation has already been identified in cancers such as hepatocellular carcinoma and lung cancer [53,54]. Recent studies suggest that in the absence of close regulation, IL-22 also promotes colon carcinogenesis via STAT3 activation [55]. To this end, Huber et al. recently reported that IL-22 is involved in colitis associated colon cancer in a dual manner. On the one hand, IL-22 deficiency can delay tissue repair, thereby sustaining inflammation and leading to tumor development. On the other hand, high levels of IL-22 in *Il22bp*^−/−^ mice may prolong the regenerative program and promote colon carcinogenesis [56]. Clinical data show an association between high serum IL-22 levels and resistance to chemotherapy in patients with colorectal cancer, an observation which was further confirmed in vitro [57,58]. Recently, Kryczek et al. suggested that IL-22 can enhance colon cancer stemness. Specifically, IL-22 was found to activate STAT3 in human colon cancer cells, resulting in the expression of the H3K9-specific *N*-methyltransferase DOT1L, which subsequently induced the expression of core stem cell genes such as SOX2, NANOG, and POU5F1. This pathway promoted colon carcinogenesis and the expression of the implicated genes was associated with poor patient prognosis [59]. IL-22 is known to promote and sustain the survival of normal intestinal stem cells in mice [60]. Therefore, a similar function resulting in maintaining the cancer stem cell niche is likely to be one of the main contributions of IL-22 to colorectal carcinogenesis. Other studies showed that high expression of RORγt, which is essential for IL-22 production, and IL-17A, which is commonly found in association with IL-22, indicates poor prognosis in patients with colorectal cancer [39].

### 3.3. Effects of TNF-α on Wound Healing

Upon inflammation-induced disruption of the intestinal mucosal barrier, intestinal epithelial cells (IECs) need to orchestrate the process of tissue restitution and regeneration. To this end, a crosstalk between epithelial and immune cells needs to take place. TNF-α, an important pro-inflammatory cytokine, is one of the implicated molecules, playing a key role during inflammation and subsequent wound healing [61]. Two forms of TNF-α, the soluble and its precursor, transmembrane one, participate in the inflammatory process. Transmembrane TNF-α acts via cell-to-cell contact, whereas soluble TNF-α performs its biological effects at distant sites from the TNF-α-secreting cells [62]. Transmembrane TNF-α acts both as a ligand by interacting with TNF-α receptors as well as a receptor that receives and transfers signals back into the same transmembrane TNF-α-expressing cells [63]. During intestinal inflammation, TNF-α is produced by immune cells such as T_H_17 cells, stromal cells, and IECs and subsequently interacts with the latter via two receptors: TNF-R1 (TNFRSF1A) and TNF-R2 (TNFRSF1B). TNF-α signaling mediates the production of inflammatory molecules, regulates cell survival, proliferation, and death, and affects epithelial wound healing. Wound healing is a complicated process involving cellular migration, re-differentiation, and proliferation. TNF-α/TNF-R2 signaling mediates epithelial migration and enhances the survival and proliferation of IECs [64]. Furthermore, TNF-α promotes intestinal wound healing by protecting against epithelial apoptosis via ErbB pathway activation. TNF-α also enhances re-epithelialization and thus wound healing by promoting the FGF-7 production. TNF-α induced effects depend on concentration and duration of exposure. Specifically, low levels of TNF-α promote inflammation and stimulate the production of macrophage-derived growth factors facilitating wound healing. However, long exposure to high levels of TNF-α can have a negative impact on healing, as TNF-α can lead to reduced production of ECM components while promoting the synthesis of metalloproteinases (MMP-1, MMP-2, MMP-3, MMP-9, MMP-13, and MT1-MMP). In line with this, TNF-α levels are increased in chronic wounds. Infection can further promote TNF-α accumulation by prolonging inflammation.

A frequent consequence of IBD and especially ulcerative colitis is colon cancer. Although initially, TNF-α was considered to serve as a tumor-suppressive factor, due to its conditional pro-apoptotic function, it was recently found to promote colitis-associated tumor development by linking inflammation and cancer [65,66,67]. TNF-α can interact strongly with intestinal epithelial cells, which express high levels of TNFR1, leading to the activation of NF-κB-dependent oncogenic pathways. Recently, Popivanova et al. identified TNF-α as a key factor for the development of colitis-related colon cancer. Specifically, upon DSS/AOM-induced colon carcinogenesis, the expression of TNF-α and the intestinal recruitment of leukocytes expressing the main TNF receptor, namely TNF-Rp55, were boosted, resulting in the formation of several intestinal tumors. Absence of TNF-Rp55 or specific blocking of TNF-α led to reduced mucosal injury and inflammation followed by decreased tumor formation [67]. In other studies, TNF-α deficiency was associated with severe colitis and cancer along with increased blood levels of IL-6, IFN-γ, and IL-17A. Similarly, recent studies showed that TNF-α mRNA expression was increased in colorectal tumors compared to surrounding healthy intestinal tissue. Interestingly, TNF-α was overexpressed in Stage III and IV tumors, suggesting that high TNF-α expression in tumor cells can be associated with advanced stages of carcinogenesis [68]. Additionally, a genetic link between TNF-α and colorectal cancer has been identified recently. Furthermore, TNF was found to play a key role in the colon cancer promoting effect of obesity [69]. Finally, TNF may also enhance metastasis by promoting epithelial to mesenchymal transition (EMT) in colorectal cancer [70].

## 4. Control of T_H_17 Cells

T_H_17 cells were originally discovered in an autoimmune setting. In a mouse model of multiple sclerosis, experimental autoimmune encephalomyelitis (EAE), and a mouse model of arthritis, both formerly linked to an uncontrolled T_H_1 response, it was discovered that not IL-12, the cytokine driving T_H_1 differentiation, but IL-23 was essential for disease development [71,72]. From in vitro studies, it has been shown that IL-23 can induce the production of IL-17 from effector and memory T cells [73]. Finally, in 2005, Langrish et al. demonstrated that IL-23 induced IL-17 producing T cells displayed stronger pathogenic properties in EAE than T_H_1 cells, and that these T_H_17 cells have a distinct gene-expression profile [74]. Besides the described protective properties of T_H_17 cells, this cell type is apparently also associated with autoimmune diseases, chronic inflammatory conditions, and carcinogenesis. Multiple sclerosis, rheumatoid arthritis, IBD, and psoriasis are amongst the diseases with a strong T_H_17 cell involvement [75,76,77,78]. Additionally, as discussed above, uncontrolled IL-22, IL-17, and TNF-α level can promote carcinogenesis. Therefore, the immune system needs control mechanisms to keep T_H_17 cells in check. There are several layers to control T_H_17 cells, which are discussed in the next sections.

### 4.1. T_H_17 Cell Differentiation

The first level of control already occurs under physiological conditions by regulating T_H_17 cell differentiation. In the past decade, intensive studies have further elucidated the signaling pathways leading to the differentiation of T_H_17 cells. Noteworthy, IL-23 signaling is not essential for the induction of T_H_17 cells from naïve T cells, since naïve T cells only express very low amounts of IL-23R [79]. Nonetheless, IL-23 signaling is crucial for the terminal differentiation, expansion, and maintenance of T_H_17 cells. IL23R-deficient T_H_17 cells fail to maintain IL-17 expression in vivo and cannot induce EAE [80]. IL-6 signaling can induce the expression of IL-23R, a crucial step in the early priming phase of T_H_17 cells. This leads to the activation of STAT3. Translocation of phosphorylated STAT3 dimers to the nucleus results in induction of T_H_17-related genes such as *Rorc*, *Il17*, and also *Il23r*. The induction of *Rorc* (encoding RORγt) is indispensable for T_H_17 cell development [81,82,83]. RORγt is the master transcriptional regulator of T_H_17 cells, demonstrated by the absence of IL-17 producing T cells in RORγt-deficient mice [17].

TGF-β is another cytokine contributing to the development of T_H_17 cells, even though its part in this process is still controversial. In low concentrations, TGF-β can inhibit T_H_1 and T_H_2 differentiation by inhibiting IL-2 dependent STAT5 activation and expression of T-bet and GATA3, the master regulators of T_H_1 and T_H_2, respectively [84]. Nonetheless, higher concentrations of TGF-β result in downregulation of IL-23R and consequently counter regulate T_H_17 cell expansion [79]. Additionally, in 2010, it was demonstrated that T_H_17 cells can occur in the absence of TGF-β signaling in the gut mucosa in vivo [85]. On the contrary, TGF-β signaling can induce the differentiation of inducible regulatory T cells (pTreg). TGF-β is dispensable for T_H_17 cell differentiation but non-redundant for the induction of pTregs [85]. TGF-β signaling induces both FOXP3, the master transcription factor of Treg cells, and RORγt expression. However, in the absence of IL-6 signaling, FOXP3 abrogates the effects of RORγt [79,86,87]. Additionally, IL-2 signaling can both enhance FOXP3 expression and induce STAT5, which leads to impaired binding of STAT3 to IL-17 related genes and inhibits T_H_17 cell differentiation [88,89,90]. In the absence of pro-inflammatory cytokines, such as IL-6 or IL-1β, TGF-β favors the development of regulatory T cells to maintain immune homeostasis. T_H_17 cells and Treg cells are cell subsets with opposite functions for the immune system, however, they share common pathways for their differentiation. This close relationship demonstrates the important and delicate balance the immune system has to maintain to guarantee immune homeostasis in the presence of foreign antigens from commensal microorganisms and food and to guarantee effective protection against pathogens. Besides TGF-β, the cytokine IL-27 is known to negatively regulate T_H_17 cell induction. IL-27 signaling inhibits the expression of RORγt and therefore suppresses T_H_17 cell differentiation [91]. On the other hand, IL-27 can induce the differentiation of another regulatory T cell subset, type one regulatory T cells (T_R_1). These cells are characterized by high expression levels of IL-10, they, however, lack FOXP3 expression [92,93]. In summary, the differentiation of the two major regulatory T cell subsets is inversely related with T_H_17 cell induction, a phenomenon not known for other effector T cell subsets, such as T_H_1 and T_H_2.

Furthermore, IL-1β is important for the differentiation of T_H_17 cells, which was already established in human T cells in 2007 [94,95]. Unlike TGF-β signaling, it was demonstrated by using mice that IL-1β signaling was crucial for T_H_17 cell induction in all tissues in vivo [96]. IL-1β signaling has multiple effects on the differentiation of T_H_17 cells. However, one essential consequence is the induction of the transcription factor IRF4, which is strictly needed for RORγt expression [97]. Interestingly, T_H_17 cells, which differentiated in the absence of TGF-β signaling, seem to have an altered phenotype. Since TGF-β is required for the suppression of T-bet expression in T_H_17 cells, IL-1β induced T_H_17 cells are also T-bet positive and co-express IFN-γ, the signature cytokine of T_H_1 cells [85]. These IFN-γ producing T_H_17 cells are frequently linked with the occurrence of autoimmune disease such as multiple sclerosis [98].

Finally, the microbiota plays an important role in T_H_17 cell differentiation. Under physiological conditions, T_H_17 cells are most abundant in the lamina propria of the small intestine [17] due to the presence of intestinal microbiota. Studies demonstrated that germ free mice have dramatically reduced levels of T_H_17 cells, which can be induced by colonialization with conventional microbiota [99]. SFB were identified as contributing to the expansion of T_H_17 cells in the small intestine due to the induction of serum amyloid A (SAA), which can stimulate DCs to release IL-6 and IL-23 and finally promote T_H_17 cell differentiation [16]. Another effect of the microbiota is the induction of IL-1β, further contributing to T_H_17 cell development [100].

Last, but not least, ligands for the aryl hydrocarbon receptor (AHR) also derive from diet or are products of the intestinal microbiota. AHR is another transcription factor which plays a non-redundant role for T_H_17 cell biology. It has been reported that AHR can promote T_H_17 cell differentiation and is already highly expressed during the early T_H_17 cell polarization [101,102]. However, contradicting studies reported an increase in T_H_17 cells in AHR-deficient mice, especially in the small intestine, demonstrating that AHR is not essential for T_H_17 cell development [103]. Nonetheless, AHR expression is crucial for IL-22 secretion by T_H_17 cells and therefore important for some tissue regenerative functions of T_H_17 cells [101,104,105].

In conclusion, T_H_17 cell differentiation is strongly influenced by the cytokine environment in different tissues of the body and the presence or absence of environmental factors such as microbiota. In the last decade, immense efforts have been made to understand the regulation of T_H_17 cell induction. Environmental factors, such as microbiota or diet, can directly or indirectly via DCs influence the development and phenotype of T_H_17 cells. Various cytokines are involved in the differentiation of T_H_17 cells and a complex transcriptional network orchestrates this process. Understanding the whole picture could facilitate the design of new therapeutic strategies targeting T_H_17 cells.

### 4.2. Regulation of T_H_17 Cells Expansion

A second mechanism is to control the expansion of T_H_17 cells via regulatory T cells. Upon tissue damage and infections with extracellular bacteria or fungi, T_H_17 cell immunity is strictly required. A pro-inflammatory environment favors the differentiation of T_H_17 cells over regulatory T cells. Nonetheless, this immune response must be regulated to prevent the onset of chronic inflammatory conditions. Likewise, during wound healing the inflammatory phase needs to be ended. Very little is known about the direct role of T_H_17 cells in the inflammatory phase. However, it was demonstrated that the absence of regulatory T cells results in decreased inflammation resolution after myocardial infarct injury and delayed wound healing in skin, further underlining the non-redundant role of CD4^+^ T cells in regulation of wound repair and regeneration [106,107]. Regulatory T cells, both Treg cells and T_R_1 cells, are key to terminate T_H_17 cell associated immune responses by suppressing the expansion of T_H_17 cells. Both regulatory T cell subsets can suppress T_H_17 cell expansion in vivo [108,109]. A major suppressive mechanism of regulatory T cells is the release of anti-inflammatory factors, such as TGF-β and IL-10. IL-10 signaling is a key factor to dampen inflammatory responses. IL-10 deficiency leads to severe inflammatory diseases in humans [110]. T_H_17 cells express IL-10 receptor and can be directly controlled via IL-10 released by regulatory T cells [111]. Another effect of IL-10 signaling is the reinforcing of regulatory T cell stability [112,113]. An environment high in IL-10 will therefore directly inhibit or terminate a T_H_17 cell immune response and amplify anti-inflammatory T cell subsets.

### 4.3. T_H_17 Cell Plasticity

A third mechanism to regulate T_H_17 cell immune responses lies within the T_H_17 cells themselves. T_H_17 cells display a great plasticity depending on their cytokine environment. The acquisition of IFN-γ production occurs frequently during inflammation and is linked to disease progression in multiple human diseases [114,115,116]. T_H_17 cells can also acquire the production of the T_H_2 signature cytokine, IL-4 [117]. These cells are present in patients suffering from allergic asthma, and in mice it has been demonstrated that these IL-4 producing T_H_17 cells have greater potential to induce asthma than conventional T_H_2 cells [117,118]. However, T_H_17 cells can also acquire a regulatory phenotype. T_H_17 cells can start producing IL-10 themselves, and it has been demonstrated that these regulatory T_H_17 cells can suppress other effector T cells in vitro and display a non-inflammatory gene expression profile [119]. Finally, due to the usage of IL-17A fate-mapping mice, it was possible to prove that T_H_17 cells can completely transdifferentiate into T_R_1 cells and therefore contribute to the resolution of inflammation [120]. The key for new therapeutic approaches for inflammatory diseases may lie especially within this T_H_17 cell plasticity.

### 4.4. Regulation of IL-22 via IL-22BP

Finally, there are ways to control the activity of T_H_17 cell associated cytokines, such as IL-22. Apart from its protective characteristics, IL-22 is known to play a pathogenic role in autoimmune diseases, several cancers, and chronic liver damage. In order to successfully maintain the balance between protection and harm, endogenous mechanisms controlling the activity of IL-22 are required. IL-22 binding protein (IL-22BP, IL-22Ra2) is a soluble IL-22 receptor and inhibitor. It has been shown that IL-22BP binds to IL-22 and blocks its interaction with the membrane bound IL-22R1 in vitro using human and mouse cells. In vivo studies using mouse models have concluded that the effect of IL-22BP is dependent on the presence of IL-22, revealing a specific in vivo binding of IL-22BP to IL-22 with higher affinity than to the membrane-bound IL-22R1 [121,122,123,124]. IL-22BP is present in lymphatic organs, the gastrointestinal system, the lung, the skin, the liver, and in the female reproductive system. Normally, cellular sources of IL-22BP detected in lymphoid organs and the intestine are conventional dendritic cells (DCs), T cells, and eosinophils. It has been shown previously that endogenous IL-22BP is responsible for controlling IL-22-induced effects in the intestine. Recently, Pelczar et al. identified the role of IL-22BP in the development of IBD [125]. It was shown that T cell derived IL-22BP promotes IBD development. CD4^+^ T cells from patients with IBD were found to produce high levels of IL-22BP. Interestingly, reduced IL-22BP expression was found in intestinal CD4^+^ T cells derived from IBD patients treated with anti-TNF-α antibodies. Therefore, these studies suggest that the regulation of IL-22 by IL-22BP is crucial and may serve as a therapeutic target in diseases like IBD.

## 5. Concluding Remarks and Future Perspective

Adaptive immunity plays a non-redundant role not only for defense against infections, but also for wound healing, tissue regeneration, or carcinogenesis. However, adaptive immunity has both beneficial and potential pathogenic characteristics. The immune system performs a constant balancing act to maintain beneficial properties over pathogenic ones. T_H_17 cells have been a main focus of immunological research since their discovery in 2005. As described above, T_H_17 cells and T_H_17 cell related cytokines can act in a beneficial fashion during wound repair and regeneration, but they can also cause chronic inflammation and carcinogenesis. Therefore, the immune system developed several control mechanisms to regulate T_H_17 cell mediated immunity. However, failure of these control mechanisms results in chronic inflammatory diseases and cancer. Thus, new therapeutic strategies targeting T_H_17 cells have been a main focus of clinical research in recent years. Nonetheless, there are many remaining open questions regarding the involvement and regulation of T_H_17 cells during tissue regeneration and wound healing. It has long been known that αβ CD4^+^ T cells infiltrate the wound bed, however, recent research lacks detailed analysis of these cells during wound healing. T_H_17 cells are especially of great interest in this matter, since they can attract neutrophils and other innate immune cells and induce anti-microbial peptides from epithelial cells. Therefore, T_H_17 cells could help to prevent spreading infections in a wound. Additionally, T_H_17 cell associated cytokines such as IL-22 can promote epithelial cell proliferation. However, the basic question if the inflammatory phase during wound healing is altered in the absence of T_H_17 cells has not been fully addressed yet. A detailed characterization of T_H_17 cell immune responses during wound healing in different tissues is needed. T_H_17 cells are most abundant in the intestine and other barrier organs such as lung and skin, therefore it can be assumed that T_H_17 cells are most important during wound healing in these organs. Besides the beneficial properties of T_H_17 cells, these cells are strongly associated with chronic inflammatory conditions. Prolonged inflammation is a common hallmark for chronic wounds, which represent an increasing health threat and a therapeutic challenge. Whether or not an uncontrolled T_H_17 cell immune response plays an important part during this process is unknown so far. However, if this is the case, it is crucial to understand control mechanisms of T_H_17 cells that could allow the reprogramming of the immune system so that this chronic inflammatory stage can be resolved. Establishing a better understanding of this process and the underlying mechanisms could potentially facilitate the design of new therapeutic approaches for a wide variety of diseases including infections, cancer, and immune mediated inflammatory diseases.
